# Hoarseness as the initial presentation of multiple myeloma

**DOI:** 10.1002/ccr3.7322

**Published:** 2023-05-12

**Authors:** Jules Colebunders, Stig Hellemans, Melek Ahmed, Sasha Libbrecht, Sévérine de Bruijn

**Affiliations:** ^1^ Faculty of Medicine and Health Sciences University of Antwerp Wilrijk Belgium; ^2^ Department of Anatomic Pathology Antwerp University Hospital Edegem Belgium; ^3^ Department of Haematology Antwerp University Hospital Edegem Belgium

**Keywords:** dysphonia, hoarseness, laryngeal mass, multiple myeloma, thyroid cartilage, tumor

## Abstract

**Key Clinical Message:**

Myeloma of the thyroid cartilage is a rare but important differential diagnosis of a laryngeal mass. Although hoarseness as the initial presenting symptom in multiple myeloma is extremely rare, a clinician should always consider it.

**Abstract:**

Multiple myeloma (MM) is a malignant plasma cell disorder characterized by an uncontrolled proliferation of monoclonal plasma cells. Although the clinical presentation at diagnosis can be quite variable, thyroid cartilage infiltration in MM is rare. Here we discuss a 65‐year‐old Caucasian male presenting to the ENT doctor with continuous hoarseness for 3 months. The initial clinical examination showed a tangible mass at the left lymph node level II–III. Further examination with fiber‐optic laryngoscopy showed a bulging of the aryepiglottic and ventricular fold. Neck and chest CT scan revealed multiple osteolytic bone lesions in addition to the large lesion in the left thyroid cartilage. Laboratory work‐up, PET‐CT scan and biopsy of the thyroid cartilage were performed and eventually all confirmed the presence of a new diagnosis of IgA kappa MM. The patient was referred to the department of hematology to start with chemotherapy.

## Graphical abstract

This case report presents a rare case of multiple myeloma in which hoarseness was the initial presenting symptom.

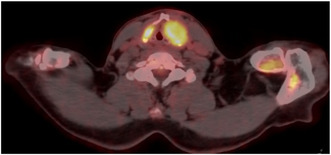

## 1 INTRODUCTION

Multiple myeloma (MM), a malignant plasma cell disorder of the post‐germinal center B‐cells in the bone marrow, is the second most common hematological malignancy in developed countries and accounts for around 1% of all cancers worldwide.^1^, ^2^, ^3^ In almost all patients, MM initially present as monoclonal gammopathy of undetermined significance (MGUS) and is characterized by an asymptomatic, uncontrolled proliferation of monoclonal plasma cells without any evidence of end‐organ damage. The plasma cells produce nonfunctional, intact immunoglobulins or immunoglobulin chains which can be detected in the serum and form the basis of the initial diagnosis.^4^ Specific criteria for progression to and the diagnosis of MM are defined by the International Myeloma Working Group (IMWG) and include validated biomarkers in addition to existing requirements of attributable CRAB features (hypercalcemia, renal failure, anemia, and bone lesions).^5^ In uncommon cases, the plasma cell neoplasm may arise within the soft tissues, called extramedullary plasmacytomas (EMP).^6^

The clinical presentation of MM at diagnosis is in most cases quite variable and non‐specific.^1^ Based on a retrospective single‐institution study of 1027 patients, the most common symptoms at presentation were: anemia (73%), bone pain (58%), elevated creatinine level (48%), fatigue (32%), hypercalcemia (28%), and weight loss (24%).^7^

In this case report we discuss a 65‐year‐old Caucasian male who presented with hoarseness as the initial symptom of MM.

## 2 CASE REPORT

A 65‐year‐old male presented to the ear, nose, and throat (ENT) department at the Antwerp University Hospital. The patient reported continuous hoarseness since 3 months which initially started as a tickling cough. There were no periods of complete aphonia. He also complained of sternal pain radiating to the right ribcage, triggered by coughing and sneezing. The intake of solid and liquid substances was preserved and no dysphagia for liquids or solids was reported. The patient showed no weight loss, night sweats or fever. At the time of presentation, the patient had no significant medical history or known allergies and did not take any medication. He had a history of smoking (five pack years, 40 years ago) and alcohol use was limited to two to three units per day. The hoarseness made it impossible to work as he was a professional voice user.

**FIGURE 1:**
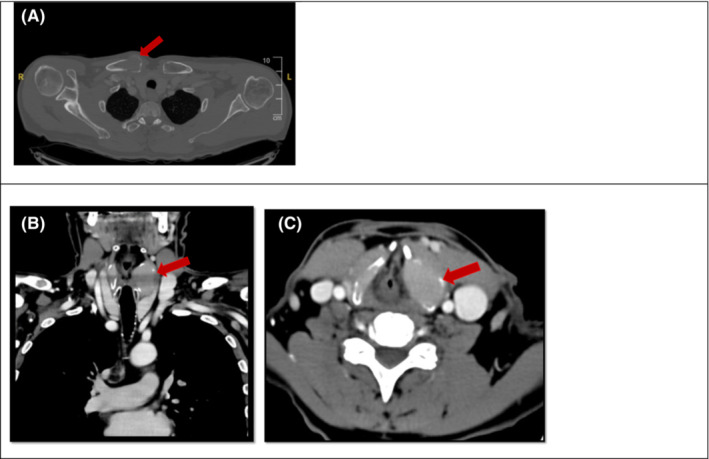
Multislice CT‐scan of neck and chest. (A) Osteolytic bone lesion in the medial right clavicula. (B,C) Multiple soft tissue density masses in the right and left thyroid cartilage. The largest is on the left with a locoregional mass effect (30 × 23 × 33 mm).

On the clinical examination, a tangible mass was discovered in the neck at the left lymph node level II–III. Except for distinct hoarseness, no other abnormalities were observed. To further investigate the pharynx and larynx, a fiber‐optic laryngoscopy was performed. A bulging of the left aryepiglottic fold and ventricular fold was observed. Stroboscopy showed asymmetry of the vocal cords with irregular movement and suboptimal glottal closure. It is unclear whether the hoarseness is due to nerve damage or physical obstruction or a combination of both. Considering there was a subtle mass tangible at palpation and a clear bulging of the aryepiglottic and ventricular fold, a CT scan of neck and chest was performed showing diffuse osteolytic bone lesions of which some had an extraosseous extension (Figure 1A). Furthermore, multiple lesions were found in the thyroid cartilage of which the largest lesion on the left side with extensive extraosseous extension (Figure 1B,C).

To further examine the cause, a more extensive work‐up with laboratory analysis (including protein electrophoresis and immunofixation), PET‐CT scan and an ultrasound‐guided Tru‐Cut biopsy of the right clavicula and the left thyroid cartilage were performed. Lab results showed an M‐peak of 57,3 g/L on protein electrophoresis with IgA protein level of 49.84 g/L, hypercalcemia, renal insufficiency and a mild anemia, all suggestive of MM with high tumor burden (Table 1).

**TABLE 1:** Overview of the most important laboratory results with diagnosis.

**Complete blood count**	Hemoglobin	11.6 mg/dL	**Protein electrophoresis**	Albumin	34.7 g/L
	Thrombocytes	228 × 10^9^/l		Alpha‐1	2.8 g/L
	Leukocytes	12.3 × 10^9^/l		Alpha‐2	5.5 g/L
**Biochemistry**	eGFR CKD‐EPI	44 mL/min		Beta‐1	3.1 g/L
	Creatinine	1.61 mg/dL		Beta‐2	59.0 g/L
	Lactate dehydrogenase	193 U/L		Gamma	2.9 g/L
	Potassium	4.4 mmoL/L		M‐peak	53.3 g/L
	Calcium	2.28 mmoL/L	**Immune fixation**	Anti‐IgA	Positive
	ß2 microglobulin	10.68 mg/L		Anti‐IgG	Negative
**Immunoglobins**	IgA	49.84 g/L		Anti‐IgM	Negative
	IgM	0.09 g/L		Anti‐kappa	Positive
	IgG	2.99 g/L		Anti‐lambda	Negative
	Free light chains kappa	1240.0 g/L			
	Free light chains lambda	3.9 g/L			

**FIGURE 2:**
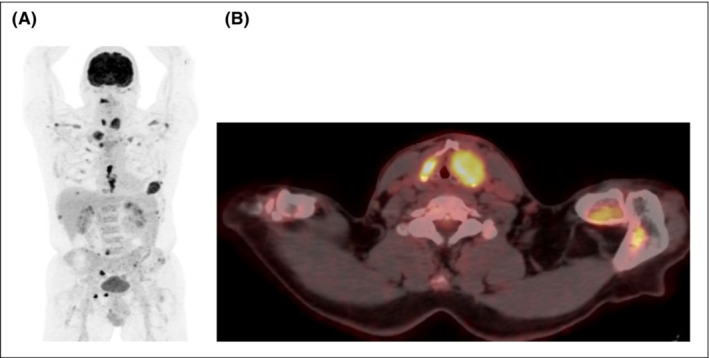
18‐FDG PET‐CT. Multiple foci of strongly FDG‐avid lesions can be observed throughout the skeleton and bilateral in the thyroid cartilage (L > R). No other zones with increased tracer activity can be detected. (A) Coronal view of maximum intensity projection. (B) Axial cervical section of PET‐CT.

**FIGURE 3:**
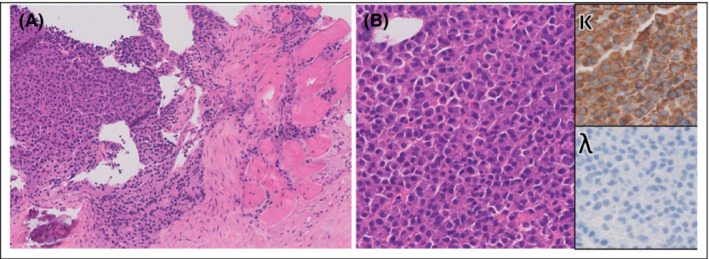
Tru‐Cut biopsy of thyroid cartilage: Several cores showing a diffuse infiltration of a mild to moderately atypical plasma cells. No pre‐existing thyroid cartilage was recognized. (A) Atypical plasma cells (on the left) showing destructive infiltration of perilaryngeal fat and striated muscle (on the right). (B) Close of up the atypical plasma cell infiltrate showing kappa light chain restriction on immunohistochemistry (inlet). Magnification: (A) 20×, (B) 40×.

Moreover, PET‐CT scan demonstrated multiple intense foci in the skeleton and bilateral in the thyroid cartilage suspicious for a malignancy (Figure 2). A Tru‐Cut biopsy was taken, showing diffuse tissue invasion by a plasma cell neoplasia with kappa light chain restriction, conforming the presence of IgA kappa MM (Figure 3).

Thereafter, the patient was urgently referred to the hematology department to start with chemotherapy after performing a bone marrow biopsy and additional lab analysis. The bone marrow was invaded by an atypical population of plasma cells with kappa light chain restriction, making up 60%–65% of bone marrow cellularity. Cytogenetics showed no high‐risk chromosomal abnormalities and his R‐ISS score^8^ was stage II, due to a ß2 microglobulin level of 10,68 mg/L and serum albumin level of 34.7 g/L, which corresponds with a median progression‐free survival of 42 months. However, the extramedullary nature of the disease is not taken into account in the scoring system of the R‐ISS.

As the patient was transplant‐eligible we started with induction chemotherapy consisting of bortezomib, cyclophosphamide and dexamethasone (VCD) once weekly, with the purpose of autologous stem cell transplantation as consolidation after four 28‐day cycles of VCD. It was expected that the dysphonia would get better with treatment.

## 3 DISCUSSION

In most patients with MM the plasma cell proliferation is restricted to the bone marrow, however a subset develops soft‐tissue plasmacytomas, whereby clonal plasma cells escape and are found outside the bone marrow.^9^ Hoarseness, as the initial presenting symptom in patients with MM is exceedingly rare but can be the result of EMP with involvement of the thyroid cartilage. Only a few cases of EMP of the thyroid cartilage have been reported in the literature so far, and as with other extramedullary disease, it is associated with a poor prognosis.^10^, ^11^, ^12^, ^13^, ^14^, ^15^, ^16^ Two possible mechanisms for thyroid cartilage involvement have been suggested: (1) Osseous metaplasia of the cartilage into bone marrow and the subsequent proliferation of plasma cells or (2) direct invasion by an adjacent soft tissue plasmacytoma into the thyroid cartilage. The exact mechanism, however, remains elusive.^10^

Although the most common cause of a thyroid cartilage mass is a squamous cell carcinoma, around 80% of EMPs are also located in the head and neck region, making this an uncommon but important differential diagnosis.^16^

While hoarseness as the initial presenting symptom in MM is extremely rare, a clinician should always consider it, especially in the presence of systemic symptoms like hypercalcaemia, unexplained renal insufficiency or osteolytic bone lesions.

## AUTHOR CONTRIBUTIONS

**Jules Colebunders:** Conceptualization; methodology; writing – original draft. **Stig Hellemans:** Conceptualization; methodology; writing – original draft. **Melek Ahmed:** Investigation; supervision; writing – original draft. **Sasha Libbrecht:** Investigation; supervision; writing – original draft. **Sévérine de Bruijn:** Supervision; writing – original draft.

## FUNDING INFORMATION

None of the authors involved in the writing of this case report received financial support from any party in relationship to content of this manuscript that might influence the authors opinion or editorial independence in the present or recent past.

## CONFLICT OF INTEREST STATEMENT

The authors have nothing to disclose and indicate no potential conflict of interest.

## CONSENT

Written informed consent was obtained from the patient to publish this report in accordance with the journal's patient consent policy.

## Data Availability

The data that support the findings of this study are available from the corresponding author upon reasonable request.

## References

[1] Gerecke C , Fuhrmann S , Strifler S , Schmidt‐Hieber M , Einsele H , Knop S . The diagnosis and treatment of multiple myeloma. Dtsch Arztebl Int. 2016;113(27–28):470‐476. doi:10.3238/arztebl.2016.0470 27476706 PMC4973001

[2] Meuleman N , Caers J , Fostier K , Vlummens P , Pans S . Guidelines of the Belgian hematology society for imaging in multiple myeloma. Belg J Hematol. 2021;12(8):338‐343.

[3] van de Donk N , Pawlyn C , Yong KL . Multiple myeloma. Lancet. 2021;397(10272):410‐427. doi:10.1016/S0140-6736(21)00135-5 33516340

[4] Korde N , Kristinsson SY , Landgren O . Monoclonal gammopathy of undetermined significance (MGUS) and smoldering multiple myeloma (SMM): novel biological insights and development of early treatment strategies. Blood. 2011;117(21):5573‐5581. doi:10.1182/blood-2011-01-270140 21441462 PMC3316455

[5] Rajkumar SV , Dimopoulos MA , Palumbo A , et al. International myeloma working group updated criteria for the diagnosis of multiple myeloma. Lancet Oncol. 2014;15(12):e538‐e548. doi:10.1016/S1470-2045(14)70442-5 25439696

[6] Caers J , Paiva B , Zamagni E , et al. Diagnosis, treatment, and response assessment in solitary plasmacytoma: updated recommendations from a European expert panel. J Hematol Oncol. 2018;11(1):10. doi:10.1186/s13045-017-0549-1 29338789 PMC5771205

[7] Kyle RA , Gertz MA , Witzig TE , et al. Review of 1027 patients with newly diagnosed multiple myeloma. Mayo Clin Proc. 2003;78(1):21‐33. doi:10.4065/78.1.21 12528874

[8] Sonneveld P , Avet‐Loiseau H , Lonial S , et al. Treatment of multiple myeloma with high‐risk cytogenetics: a consensus of the international myeloma working group. Blood. 2016;127(24):2955‐2962. doi:10.1182/blood-2016-01-631200 27002115 PMC4920674

[9] Blade J , Beksac M , Caers J , et al. Extramedullary disease in multiple myeloma: a systematic literature review. Blood Cancer J. 2022;12(3):45. doi:10.1038/s41408-022-00643-3 35314675 PMC8938478

[10] Singh K , Kumar P , Pruthy R , Goyal G . Multiple myeloma presenting as thyroid Plasmacytoma. Indian J Med Paediatr Oncol. 2017;38(4):552‐554. doi:10.4103/ijmpo.ijmpo_43_16 29333030 PMC5759082

[11] Kumar N , Pandey AN , Kashyap R , Agarwal G . Extramedullary relapse in a case of multiple myeloma involving the thyroid cartilage: case report and review of literature. Indian. J Surg Oncol. 2011;2(4):313‐315. doi:10.1007/s13193-011-0097-z PMC3338136 23204788

[12] Aslan I , Yenice H , Baserer N . An indolent cours of multiple myeloma mimicking a solitary thyroid cartilage plasmacytoma. Eur Arch Otorhinolaryngol. 2022;259:84‐86. 10.1007/s004050100399 11954938

[13] Gross M , Eliashar R , Petrova P , Goldfarb A , Sichel JY . Neck mass as primary manifestation of multiple myeloma originating in the thyroid cartilage. Otolaryngol Head Neck Surg. 2002;126:326‐328. 11956544 10.1067/mhn.2002.123045

[14] Sosna J , Slasky BS , Paltiel O , Pizov G , Libson E . Multiple myeloma involving the thyroid cartilage: case report. Am J Neuroradiol. 2022;23:316‐318. PMC7975273 11847062

[15] Van Dyke CW , Masaryk TJ , Lavertu P . Multiple myeloma involving the thyroid cartilage. Am J Neuroradiol. 1996;17:570‐572. 8881256 PMC8337979

[16] You WS , Bhuta S . Myeloma of laryngeal cartilage: literature review and case study. Ear Nose Throat J. 2021;100(2):NP114‐NP119. doi:10.1177/0145561319861379 31284752

